# Sensor-Based and VR-Assisted Visual Training Enhances Visuomotor Reaction Metrics in Youth Handball Players

**DOI:** 10.3390/s26082555

**Published:** 2026-04-21

**Authors:** Ricardo Bernárdez-Vilaboa, Juan E. Cedrún-Sánchez, Silvia Burgos-Postigo, Rut González-Jiménez, Carla Otero-Currás, F. Javier Povedano-Montero

**Affiliations:** 1 Optometry and Vision Department, Faculty of Optics and Optometry, Complutense University of Madrid, 28037 Madrid, Spainjcedrun@ucm.es (J.E.C.-S.); rutgon03@ucm.es (R.G.-J.);; 2 Applied Vision Research Group, Faculty of Optics and Optometry, Complutense University of Madrid, 28037 Madrid, Spain; 3 Faculty of Medicine, Health and Sports, Universidad Europea de Madrid, 28670 Madrid, Spain; silvia.burgos@universidadeuropea.es; 4 Hospital Doce de Octubre Research Institute (i+12), 28041 Madrid, Spain

**Keywords:** virtual reality, sensor-based assessment, visuomotor performance, reaction time, human performance, sports vision, stroboscopic training

## Abstract

**Highlights:**

**What are the main findings?**

- Sensor-based reaction-time systems detected selective improvements in visuomotor performance following an integrated visual training program.
- Virtual reality–assisted and stroboscopic visual training enhanced reaction speed and accommodative dynamics without altering baseline binocular alignment.

**What are the implications of the main findings?**

- Sensor-derived metrics provide a sensitive and objective approach to quantify visuomotor adaptations in applied human performance settings.
- Integrated sensor- and VR-based visual training systems may represent time-efficient tools for objective assessment and structured visuomotor training within applied settings.

**Abstract:**

Background: Sensor-based systems and virtual reality (VR) technologies provide new opportunities for the objective, technology-driven assessment and training of visuomotor performance in applied contexts such as sport. Methods: This study examined the effects of an integrated visual training program combining stroboscopic stimulation, VR-based vergence exercises, and instrumented reaction-light tasks in adolescent handball players. Twenty-eight adolescent handball players (under-18 competitive level) completed two baseline assessments separated by six weeks, followed by a six-session training program (approximately 15 min per session) integrated into regular team practice. The intervention targeted visuomotor reaction speed, accommodative dynamics, and peripheral visual responsiveness using sensor-based and virtual reality–assisted stimuli. Results: Compared with both baseline measurements, the intervention produced selective improvements in accommodative facility (cycles per minute, cpm)—particularly near–far focusing speed—and in multiple reaction-time conditions (milliseconds, ms) involving manual and decision-based responses. Specific peripheral-field locations showed increased response scores, whereas binocular alignment, AC/A ratio, near phoria, and stereoscopic acuity remained unchanged. Conclusions: These findings indicate that technology-supported visual training protocols incorporating sensor-based reaction systems and VR stimuli were associated with measurable adaptations in dynamic visuomotor processing while preserving fundamental binocular vision parameters.

## 1. Introduction

Handball is a fast-paced invasion sport in which players must continuously interpret complex visual information to make rapid and accurate decisions. In this context, sensor-based assessment and training technologies offer valuable tools to objectively quantify visuomotor demands and adaptations during performance. Actions such as anticipating an opponent’s movement, locating teammates under pressure, and executing precise passes or shots depend heavily on visual–perceptual processing. Beyond physical conditioning and technical proficiency, perceptual and cognitive demands—particularly those linked to visual processing—are increasingly recognized as key contributors to performance in team sports [1].

In recent years, increasing attention has been directed toward the role of visual and visuomotor training in enhancing athletic performance, particularly in open-skill sports that require rapid perception–action coupling. A recent systematic review by Dingirdan Gultekin et al. [2] highlighted that stroboscopic visual training can induce consistent improvements in reaction time, visuomotor coordination, and sport-specific performance outcomes across a range of athletic populations. From a broader sports vision perspective, Laby and Appelbaum [3] emphasized the relevance of structured visual training programs as complementary tools to conventional physical training, supporting their application in applied performance settings.

Experimental evidence further indicates that stroboscopic interventions can enhance visuomotor response speed and processing efficiency, as demonstrated in elite youth athletes by Hülsdünker et al. [4,5], who reported both behavioral improvements and underlying brain–behavior adaptations following short- and long-term training. More recent applied studies in open-skill sports have confirmed the transferability of these effects to ecologically valid contexts, including improvements in reactive agility and visuomotor performance in young athletes [6]. In parallel, recent reviews have reinforced the importance of vision-related functions as modifiable contributors to athletic performance, supporting the integration of targeted visual training strategies within multidisciplinary performance programs [7].

Several visual abilities are especially relevant in handball and can be objectively quantified using instrumented systems. Peripheral vision supports spatial awareness and enables players to detect stimuli outside the central visual field, an ability in which handball players often outperform athletes from more visually constrained sports [8]. Reaction speed is equally critical, as players must respond instantly to unexpected passes, defensive rotations, or shooting opportunities. Recent studies have used reaction-light systems and exergame-based platforms to both quantify and enhance these responses under controlled conditions [9,10]. Elite players also show advanced anticipatory behaviour, using subtle visual cues to predict opponents’ actions and make faster, more accurate decisions [11,12]. Collectively, these abilities contribute to visuomotor performance, a determinant of throwing accuracy, interception ability, and overall movement efficiency [13].

In fast-paced invasion sports such as handball, players must repeatedly shift their visual focus between near and far targets (e.g., ball control, teammates, opponents, and goal areas) within fractions of a second. These rapid accommodative transitions require efficient accommodative dynamics, operationally defined as the speed and flexibility with which the visual system adjusts focus between different viewing distances. In applied youth sport settings, efficient accommodative responses may facilitate faster visual information acquisition and more effective perception–action coupling during dynamic play situations. This rationale aligns with previous work highlighting the importance of specialized visual training protocols for enhancing visual processing and performance monitoring in young athletes [14].

Given the importance of these skills, interest in technology-assisted visual training has increased in recent years. Stroboscopic eyewear, which intermittently occludes vision, has been shown to enhance visual pathway efficiency and reduce electrophysiological latencies in handball and other open-skill sports [15]. In addition, research using controlled perturbations of visual input has demonstrated that stroboscopic vision can modify perceptual–motor coupling and facilitate the processing of multiple moving objects, suggesting broader benefits for decision-making under time pressure [16].

Technological tools such as reaction-light systems provide instrumented, time-resolved measures of visuomotor responses, allowing both training and quantitative assessment of reaction performance. Virtual reality (VR) and dichoptic training further enable controlled stimulation of binocular and accommodative functions in standardised and sport-specific environments, offering reproducible conditions for visual training and measurement.

Despite these developments, important gaps remain in literature. Although evidence on visual training effects reported in recent studies is growing, longitudinal data are limited, and few studies have examined whether improvements detected through sensor-derived visual and reaction-time metrics reflect meaningful changes in applied visuomotor performance. Moreover, little is known about how integrated approaches—such as stroboscopic stimulation, VR-based vergence exercises, and field-based reaction tasks—may interact to induce measurable perceptual–motor adaptations in adolescent handball players.

Recent theoretical work has highlighted the value of virtual and augmented environments for developing representative decision-making skills. According to Janssen et al. [17], VR can recreate perceptual conditions that closely resemble those encountered during competition while maintaining experimental control, supporting its use for perceptual–motor training and assessment. This perspective is consistent with earlier applications of VR in sport, which demonstrated that immersive virtual environments allow precise control of visual information and task constraints while preserving key perception–action couplings relevant to performance [18].

Understanding how integrated visual training methods affect key perceptual–motor abilities, as captured by sensor-based outcome measures, is therefore highly relevant for practitioners seeking evidence-based and time-efficient strategies to enhance visuomotor responsiveness in youth handball.

Within this context, sensor-based assessment systems combined with virtual reality environments offer a human-centered approach to quantify and enhance visuomotor and reaction-time processes in applied sports settings.

Based on the existing literature and the functional demands of handball, reaction time was defined as the primary outcome of the present study. We hypothesized that the integrated visual training program would lead to significant improvements in visuomotor reaction-time performance, given the direct stimulation of rapid visual processing, decision-making, and motor execution during the intervention.

In addition, secondary hypotheses were formulated for selected visual functions that are closely linked to visuomotor performance and were specifically targeted by the training protocol, including accommodative dynamics and peripheral visual responsiveness. Conversely, no substantial changes were expected in more stable visual parameters, such as ocular alignment or stereoscopic depth perception, which were included primarily to confirm the specificity of the intervention effects rather than to demonstrate generalized changes in visual function.

Accordingly, the aim of this study was to examine the effects of an integrated visual training program incorporating stroboscopic stimulation, VR-based vergence exercises, and instrumented reaction-light tasks on sensor-derived reaction speed and visual performance metrics in youth handball players.

## 2. Materials and Methods

### 2.1. Study Design

This study followed a quasi-experimental repeated-measures (within-subject) design including two baseline assessments separated by six weeks, followed by a six-session integrated visual training program. All players served as their own controls by completing the two pre-intervention evaluations to account for natural variability and potential learning effects in visual and visuomotor measures. This dual-baseline approach was selected to strengthen internal validity while preserving feasibility within a real-world team-sport environment.

**Figure 1:**
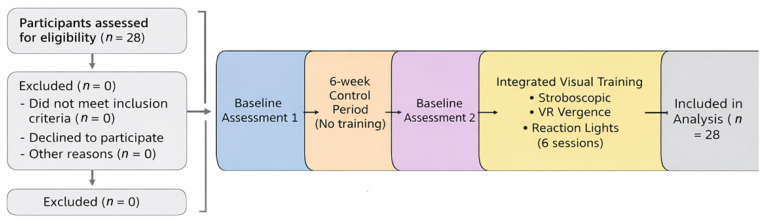
Study flow diagram summarizing assessments, control period, training sessions, and final evaluation.

A schematic flow diagram was created to summarize the structure of the study and to improve the clarity of the methodological process. All 28 participants completed two baseline assessments separated by a six-week control period without visual training. After this period, all players underwent a six-session integrated visual-training program combining stroboscopic stimulation, VR-based vergence exercises, and reaction-light drills. A post-training assessment was conducted after the intervention, and all participants were included in the final analysis. The flow of participants and the sequence of procedures are illustrated in Figure 1.

### 2.2. Participants

Twenty-eight youth handball players from Club Ikasa (Boadilla del Monte, Madrid, Spain) participated in the study. All athletes competed in under-18 categories and had a mean age of 16.85 ± 1.00 years (range: 14.99–18.44). All participants were active federation players with normal or corrected-to-normal visual acuity and no history of ocular pathology. Written informed consent was obtained from parents or legal guardians, and verbal assent was obtained from all participants.

### 2.3. Ethical Approval

The study was conducted in accordance with the principles of the Declaration of Helsinki and approved by the Research Ethics Committee (CEIm) of Hospital Clínico San Carlos, Madrid, Spain (Approval ID: 23/415-E; date of approval: 28 June 2023). As the study involved minors, written informed consent was obtained from parents or legal guardians, and assent was obtained from all participants. All personal data were processed in accordance with the European General Data Protection Regulation (GDPR, EU 2016/679).

### 2.4. Baseline Visual Assessment

Before the intervention, all athletes completed a comprehensive visual examination during two training sessions between 18:00 and 21:00. The assessment included:

- Monocular and binocular visual acuity
- Static and dynamic refractive status
- Oculomotor motility assessment
- Binocular vision (phorias, vergence ranges, near point of convergence)
- Accommodative function (facility, amplitude, response)
- Colour vision and contrast sensitivity
- Peripheral visual field screening
- Selected visual–perceptual abilities relevant to handball

After six weeks without exposure to visual training, the full protocol was repeated to establish a second baseline, used as the control comparison for the intervention.

In addition to the clinical visual examination, reaction-time assessments using the instrumented reaction-light system were also performed during both baseline sessions, following the same protocol later applied for post-intervention comparison.

### 2.5. Training Intervention

The visual training intervention was supported by a combination of sensor-based reaction systems and immersive virtual reality (VR) technology, designed to provide objective, time-resolved visuomotor stimulation under controlled conditions. All technological components were selected to ensure ecological validity, ease of integration within regular team training, and precise quantification of visuomotor responses.

The integrated visual training program consisted of six sessions, performed during regular team training, each lasting approximately 15 min. Sessions included:

(A) Reaction-light drills (10 min per session): Two motor–perceptual exercises were performed using four reaction-light devices positioned 2.95 m from the goal area.
  Reaction-time training and assessment were performed using a custom-configured reaction-light system developed for applied sport training, capable of detecting manual and foot-based responses with millisecond temporal resolution. The system automatically logs stimulus onset and response activation times and exports time-stamped data files for subsequent statistical processing, allowing objective quantification of reaction speed under both simple and choice-response conditions. Visual stimuli were presented in predefined spatial configurations to simulate sport-relevant decision-making demands, including color discrimination, stimulus–response compatibility, and rapid motor execution.
  Stroboscopic eyewear was used to intermittently restrict visual input during selected drills, thereby increasing perceptual load and promoting adaptive visuomotor processing. The stroboscopic mode alternated periods of visual availability and occlusion at predefined frequencies, with parameters adjusted to maintain task feasibility while increasing visual–perceptual challenge.

Exercise 1:

Players responded to a sequence of colour stimuli presented on a laptop screen via a custom HTML program. When a coloured circle was displayed (blue, yellow, purple, green, red), the player activated the corresponding reaction-light sensor, then sprinted to perform a throw at the goal quadrant matching that colour. Two facing lines of players alternated activation and throwing actions. Stroboscopic eyewear (Senaptec^®^, alternating-eye mode, fast duty cycle, Senaptec^®^ LLC, Beaverton, OR, USA, ) was worn by half of the athletes in each repetition.

Exercise 2:

Three reaction-light units were placed equidistantly from the goal. Players were assigned one of two colours, with lights activating in alternating random order. The player holding the ball passed to the first athlete in the opposite line only when the activated light matched the assigned colour, then sprinted to deactivate the sensor. Two versions of this drill were used:

- Version 1: players returned to their original line
- Version 2: players repositioned via the opposite side of the light array

Stroboscopic eyewear was used with an increased occlusion frequency (0.5 s open/0.5 s closed) in alternating sets.

**Figure 2:**
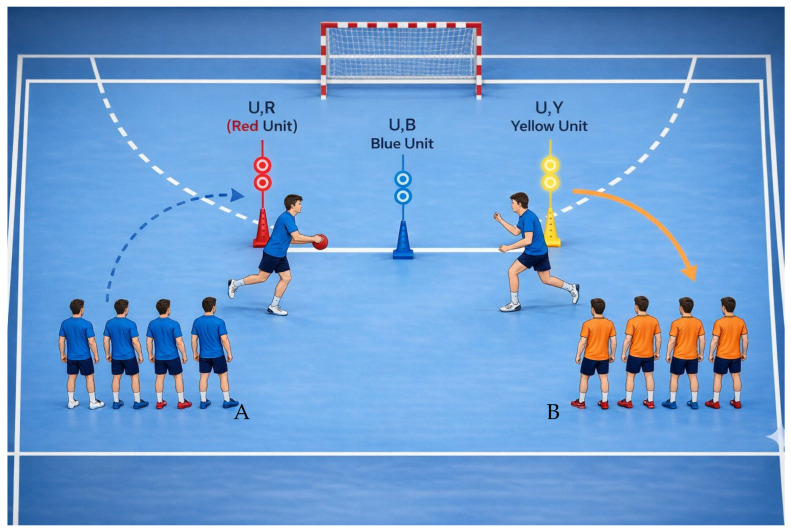
Schematic representation of the integrated visual–motor training drill (Exercise 2). Three reaction-light units were positioned equidistantly at 2.55 m from the goal area. Players responded to color-coded visual stimuli by executing rapid decision-making, passing, sprinting, and sensor deactivation actions. Two task versions were implemented: (A) Return, in which players returned to their original line after deactivating the sensor; and (B) Cross-over, involving repositioning through the opposite side of the light array. The drill was designed to simulate sport-specific perceptual–motor demands under time-constrained conditions. Blue and orange players represent the two opposing lines/teams. Arrows indicate movement direction. The star symbol indicates the sensor/light activation point.

A schematic representation of the integrated visual–motor training drill (Exercise 2) is provided in Figure 2 to illustrate the spatial configuration, task flow, and decision-making demands of the protocol.

(B) VR-based vergence training (5 min per session): The final component employed the Dicopt Home© VR (Dicopt, Madrid, Spain) platform to stimulate convergence and divergence. Diplopia difficulty was progressively increased across sessions (levels 1, 3, and 5). Convergence and divergence tasks alternated between sessions to ensure balanced stimulation of binocular function.
  VR-based training was delivered using a head-mounted display that provided immersive binocular stimulation in a controlled virtual environment. The system enabled precise manipulation of vergence demand and diplopia thresholds while maintaining stable head position and controlled viewing distance. Task difficulty was progressively increased across sessions by modifying disparity parameters, allowing systematic stimulation of convergence and divergence responses.

Additional visual materials illustrating the on-court implementation of the training drills, alternative task configurations, and VR-based training procedures are provided in the Supplementary Materials (Figures S1 and S2).

### 2.6. Outcome Measures

Primary outcome:

- Reaction speed (manual and foot-based reaction time according to the predefined protocol)

Secondary outcomes:

- Vergence facility
- Near point of convergence
- Accommodative facility (cycles per minute, cpm), used as an operational measure of accommodative dynamics, reflecting the speed and flexibility of near–far focusing responses.
- Contrast sensitivity
- Visual acuity
- Peripheral visual-field responsiveness (DIVE-derived peripheral-response scores)

All measures were obtained at Baseline 1, Baseline 2 (control), and Post-intervention. Reaction-time outcomes were derived from the automated output of the instrumented reaction-light system described above.

The alphanumeric reaction-time labels correspond to protocol-generated task identifiers automatically assigned by the reaction-light system. These codes reflect specific stimulus–response configurations and task conditions within the experimental sequence and are reported for reproducibility and traceability of system-derived outputs.

For interpretative clarity, reaction-time task codes were grouped according to their functional characteristics within the protocol. Tasks labeled with “S” corresponded to simple reaction conditions involving a single stimulus–response mapping, whereas tasks labeled with “E” represented elective (choice-based) conditions requiring stimulus discrimination and response selection. Numeric suffixes reflected predefined variations in spatial configuration and motor execution (e.g., return vs. cross-over movement patterns). These structured conditions were consistently applied across baseline and post-intervention assessments.

A detailed functional classification of the reaction-time task codes is provided in Supplementary Table S1 to facilitate transparency and reproducibility.

### 2.7. Statistical Analysis

Descriptive statistics (mean ± SD) were computed for all variables. Normality was examined using the Shapiro–Wilk test. For normally distributed variables (e.g., accommodative facility), paired *t*-tests were applied. For non-parametric variables, the Wilcoxon signed-rank test was used. Between-group comparisons (strobo vs. non-strobo) employed Mann–Whitney U tests. These comparisons were exploratory and based on alternating exposure to stroboscopic eyewear, rather than on predefined experimental groups. Statistical significance was set at *p* < 0.05. Analyses were conducted using SPSS v.27 (IBM Corp., Armonk, NY, USA).

Given the structured yet multi-contrast nature of the within-subject comparisons across predefined reaction-time task conditions, no formal adjustment for multiple comparisons was applied. The analyzed task contrasts were defined a priori according to the structured training protocol and were not generated post hoc. To enhance interpretative transparency, effect sizes were systematically reported alongside *p*-values, allowing readers to evaluate both statistical significance and practical magnitude of the observed changes. Future confirmatory studies with predefined primary outcomes and larger samples are warranted to further validate these findings.

## 3. Results

Twenty-eight youth handball players completed all evaluations. The sample had a mean age of 16.85 ± 1.00 years (range: 14.99–18.44) and showed normal or corrected-to-normal binocular visual acuity (0.96 ± 0.30). After optical compensation, all players achieved binocular VA ≥ 0.9. Weekly training load averaged 3516.33 ± 1352.99 METs, consistent with competitive youth performance (Table 1).

Stereopsis measured using the Howard–Dolman test fell within normal limits (–4.37 ± 16.32 mm). Hess test values showed no relevant horizontal or torsional deviations (median 0–2 units across positions), and assessed with the DIVE digital eye-tracking assessment system demonstrated symmetric fixation, saccadic and pursuit behavior between eyes, consistent with expected norms for this age group.

**Figure 3:**
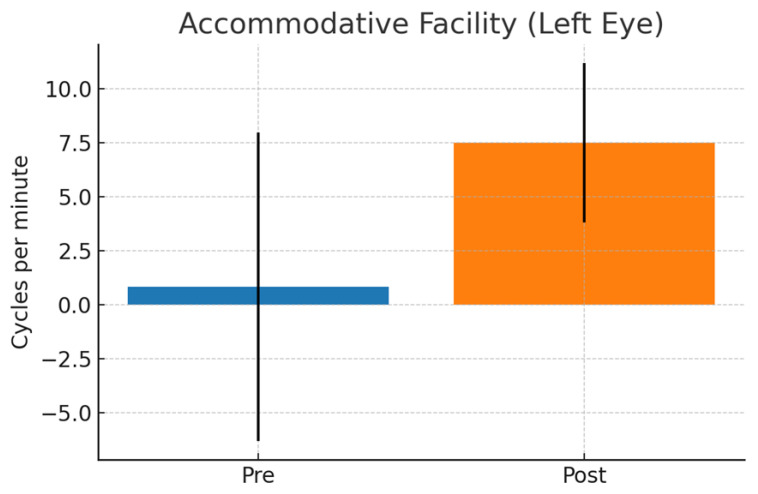
Pre–post change in accommodative facility OI.

Accommodation and binocular variables remained stable across the two pre-training evaluations. After the visual training program, a significant improvement in left-eye accommodative facility was observed (mean change = 7.50 ± 3.70 cpm; t(27) = 2.29, *p* = 0.03, d = 0.43), while no significant change was detected in the right eye. AC/A ratio and near phoria did not differ significantly after the intervention (*p* > 0.37), indicating that vergence–accommodation coupling and baseline binocular alignment remained stable (Figure 3).

**Figure 4:**
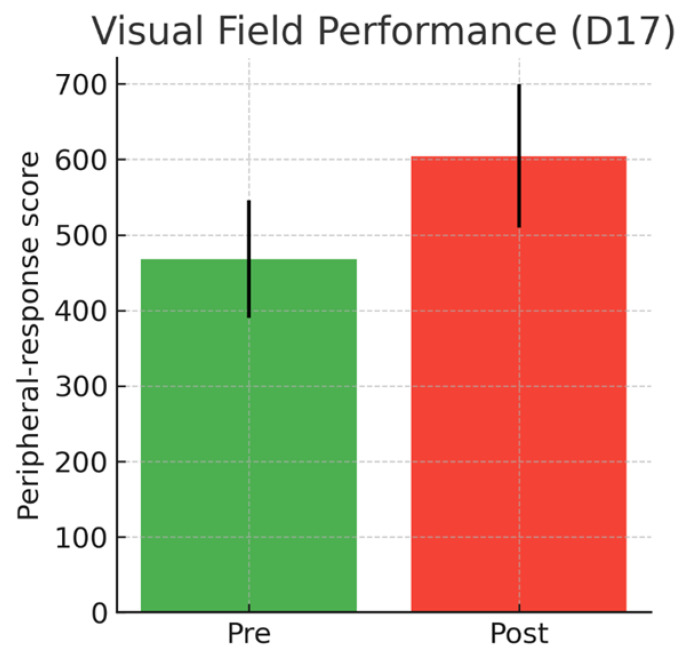
Visual-field performance at a representative peripheral position (D17). Bars represent the peripheral-response score generated by the assessment software for each condition (Pre and Post). Higher values indicate greater peripheral sensitivity. Error bars represent standard deviations.

Visual-field performance showed no significant changes during the control period. However, specific peripheral positions demonstrated significant improvement following training (*p* = 0.043–0.046), suggesting a modest enhancement of functional peripheral awareness. A representative peripheral position (D17), a predefined peripheral-field position generated by the assessment software, exhibited a significant increase in its peripheral-response score (from 467.89 ± 78.26 to 604.29 ± 95.03; *p* = 0.046), indicating greater peripheral sensitivity under the assessed task conditions (Figure 4).

Given the inter-individual variability observed in several outcome measures, additional graphical representations displaying individual data points have been included to complement the mean ± SD plots. These visualizations allow a clearer interpretation of response dispersion and individual trends underlying the group-level effects. Individual data distributions corresponding to Figure 2, Figure 3 and Figure 4 are provided in Supplementary Figures S3–S5.

**Figure 5:**
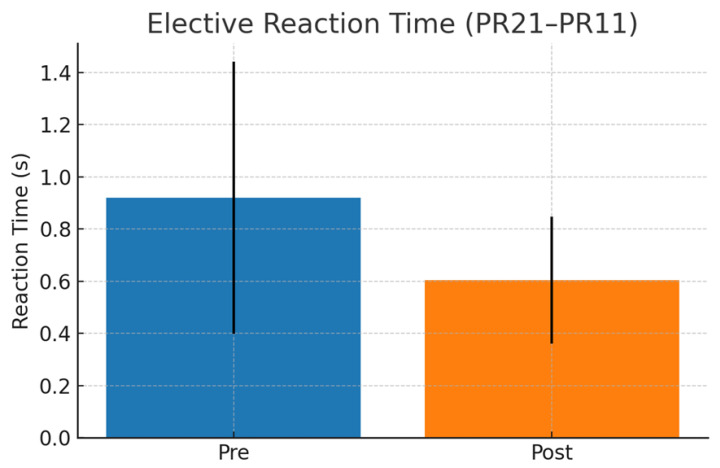
Representative elective reaction-time condition before and after the visual training program. Bars represent mean ± SD.

Reaction-time variables showed no significant differences during the control period. A representative elective reaction-time condition is shown in Figure 5. In contrast, the integrated visual training program was associated with significant changes in several predefined task-specific reaction-time conditions, particularly in manual and decision-based responses. Significant reductions were observed in S00 vs. S01 (t(27) = −2.95, *p* = 0.009, d = −0.69) and E10 vs. E11 (t(27) = −4.50, *p* < 0.001, d = −0.85). Additional significant effects were detected in S00 vs. S20 (W = 23.0, *p* = 0.011, r = 0.45), S00 vs. S21 (W = 1.0, *p* < 0.001, r = 0.86), and S20 vs. S21 (W = 1.0, *p* < 0.001, r = 0.86). Effect sizes ranged from small to large. A detailed summary of paired comparisons, including statistical tests and effect sizes, is presented in Table 2.

Overall, the integrated stroboscopic, VR-based, and reaction-light visual training program was associated with selective reductions across specific task conditions and improvements in accommodative facility, while oculomotor function, binocular alignment and basic vergence parameters remained stable. These results suggest task-specific adaptations in dynamic visual skills and visuomotor metrics under the assessed conditions.

No adverse events or dropouts were reported during the intervention period. Together, these findings support the presence of task-specific adaptations following the integrated visual training protocol, as detected by objective instrumented measures.

## 4. Discussion

The present study indicates that an integrated visual training program combining stroboscopic stimulation, virtual reality (VR)–based vergence exercises, and instrumented reaction-light tasks was associated with selective improvements in sensor-derived visuomotor metrics in youth handball players. Specifically, measurable changes were observed in accommodative facility and in predefined simple and choice reaction-time task conditions. In contrast, no meaningful modifications were detected in ocular alignment, stereoscopic depth perception, or baseline oculomotor parameters. These findings suggest that the intervention selectively influenced dynamic visual–motor processes under controlled assessment conditions, while preserving stable binocular vision functions.

These findings are consistent with previous research in sports vision training and extend existing evidence by integrating multiple technology-based components within a single program. Prior studies have shown that stroboscopic stimulation can enhance visuomotor processing and reduce response times in open-skill sports. For example, Zwierko et al. [15] reported faster visuomotor responses following stroboscopic training and attributed these effects to increased efficiency of visual information processing. The present results support this interpretation, indicating that intermittent visual occlusion may promote functional adaptations related to rapid visual processing.

An important aspect of the present intervention is its relatively short duration, consisting of six sessions of approximately 15 min integrated into regular team training. This design was intentional and aimed to maximize feasibility and compliance in an applied sports setting, where additional training loads are often difficult to implement. The program focused on high-specificity visuomotor tasks targeting reaction speed, accommodative dynamics, and peripheral processing, delivered through sensor-based and VR-assisted stimuli with high perceptual demand.

From a dose–response perspective, the observed improvements suggest that brief but targeted visual interventions may be sufficient to induce measurable adaptations in selected visuomotor parameters, particularly when assessed using sensitive, objective outcome measures. Nevertheless, it is likely that longer or more intensive training programs could produce larger or more sustained effects. Future studies should therefore examine the influence of training duration, frequency, and progression on the magnitude and persistence of visuomotor adaptations.

Similarly, the improvements in reaction-time performance observed in this study align with the work of Badau et al. [9,10], who demonstrated that training protocols incorporating reaction-light systems and exergame-based tasks can enhance response speed in team sports, including handball. Their findings highlighted the potential of light-based stimuli to improve manual reaction times, reinforcing the role of instrumented systems as effective tools for both training and objective assessment of visuomotor performance.

A further strength of the present work is that these adaptations were detected using objective, sensor-derived outcome measures, underscoring the sensitivity of instrumented assessment systems to detect selective functional changes, in line with previous evidence supporting the use of wearable and in-field sensor technologies for objective sport performance evaluation [19]. In particular, the increased response scores observed at specific peripheral-field locations suggest enhanced efficiency of peripheral visual processing. Such adaptations may allow players to detect lateral stimuli more effectively without shifting gaze away from the primary focus of action, a critical requirement in fast-paced invasion sports.

These results are in line with previous studies reporting more efficient use of peripheral vision in expert athletes compared with less experienced individuals [20,21]. Effective peripheral perception provides contextual information that supports rapid and situationally appropriate decision-making. Experimental evidence further supports this interpretation. Using multiple object tracking paradigms, Vater et al. [22] showed that participants rely heavily on peripheral vision to detect changes in dynamic environments, reinforcing the importance of peripheral processing in contexts that resemble the demands of team sports.

The observed improvement in accommodative facility may also have relevant functional implications in handball. Efficient accommodative dynamics facilitate rapid shifts of visual focus between near and far targets (e.g., ball handling, teammates, opponents, and shooting zones), supporting faster visual information acquisition and smoother perception–action coupling under time-constrained conditions. When combined with reduced reaction times, these changes may support more fluent visuomotor processing and more effective motor responses. In this regard, Poltavski and Biberdorf [23] reported that athletes with faster reaction times and more efficient focus switching exhibited superior performance, which is consistent with the functional interpretation of the adaptations observed in the present study.

The unilateral improvement observed in accommodative facility should be interpreted with caution. This inter-ocular difference is likely influenced by normal inter-ocular physiological variability between eyes, potential effects of ocular dominance, and baseline dispersion within a relatively small sample. As the present study was not specifically designed to investigate eye-specific or lateralized training effects, this finding should be considered exploratory rather than indicative of a true asymmetric adaptation. Future studies incorporating ocular dominance assessment and larger samples are warranted to clarify the consistency and functional relevance of such inter-ocular differences.

Although the current investigation focused on basic visuomotor abilities, it is plausible that these adaptations provide a functional foundation for higher-level perceptual–cognitive processes, such as anticipation and situational awareness. Elite athletes have been shown to rely on subtle visual cues and rapid scene interpretation to anticipate opponents’ actions and optimize decision-making [11]. While anticipatory skills were not directly assessed in this study, the reductions in reaction time and improvements in peripheral responsiveness observed here may provide a functional basis for the development of such abilities in applied settings.

From an applied perspective, the findings suggest that integrating sensor- and VR-based visual training components into regular practice sessions may represent a feasible strategy to enhance visuomotor responsiveness without increasing physical training load. This approach may be particularly relevant for coaches and practitioners seeking complementary interventions aimed at improving decision speed and visual processing efficiency in youth athletes.

Importantly, the intervention was designed to be embedded within regular team training sessions, requiring limited additional time and no increase in physical load. This practical integration enhances the ecological validity of the program and supports its potential adoption by coaches and practitioners as a complementary strategy to enhance visuomotor responsiveness in youth athletes [7].

The study presents several methodological strengths. The inclusion of two baseline assessments allowed control for natural variability in visual and visuomotor measures, providing a robust pre-intervention reference. In addition, the integrated training protocol combined multiple technology-assisted components rather than targeting a single visual function in isolation. The use of objective clinical, perceptual, and visuomotor measures enabled a multidimensional evaluation of intervention effects, and embedding the program within regular team training supported its practical applicability.

Several limitations should also be acknowledged. The quasi-experimental design, without an independent randomized control group, limits causal inference, although the dual-baseline approach partially mitigates this constraint. Specifically, the inclusion of two baseline assessments allowed each participant to serve as their own control, reducing the influence of natural variability and learning effects prior to the intervention.

The relatively modest sample size, typical of applied team-sport intervention studies, may limit the generalizability of the findings. However, the repeated-measures design and the use of high-resolution, objective sensor-based outcome measures enhanced sensitivity to within-subject changes. Future studies with larger samples are needed to confirm and extend these results.

The sample consisted of adolescent players from a single club, which may limit generalizability to other age groups or competitive levels. Moreover, the study did not include direct on-court performance metrics, precluding definitive conclusions regarding the competitive impact of the observed visuomotor improvements.

Future research should incorporate objective on-court performance measures and longitudinal designs to examine the persistence of the adaptations identified here. Combining sensor-based assessment systems, VR technologies, and sport-specific performance metrics may provide a more comprehensive understanding of the potential of integrated visual training approaches in handball and other team sports.

Overall, the present findings indicate that an integrated visual training approach, assessed through objective instrumented measurement systems, can produce specific visuomotor adaptations detectable under controlled task conditions in youth handball players. These results support the use of sensor-based technologies as valuable tools for the objective assessment of visuomotor performance in applied human performance settings.

## 5. Conclusions

In conclusion, the integrated visual training program combining stroboscopic stimulation, VR-based vergence exercises, and instrumented reaction-light tasks was associated with selective improvements in sensor-derived visuomotor metrics among youth handball players. The intervention enhanced accommodative facility and performance in predefined simple and choice reaction-time conditions, while binocular alignment and baseline oculomotor parameters remained stable.

These findings suggest that structured, technology-assisted visual training may selectively influence dynamic visual–motor processes under controlled assessment conditions. However, the present results should be interpreted as task-specific adaptations measured through instrumented systems rather than direct evidence of enhanced competitive match performance.

Future research incorporating randomized controlled designs, larger samples, and objective sport-specific performance metrics will be necessary to determine the transferability and long-term relevance of these adaptations in applied settings.

## Figures and Tables

**Table 1:** Baseline characteristics of the sample.

Variable	Mean ± SD/*n* (%)	Range
Age (years)	16.85 ± 1.00	14.99–18.44
Height (cm)	179.33 ± 6.7	Not applicable
Body mass (kg)	65.33 ± 0.88	Not applicable
Weekly training load (METs)	3516.33 ± 1352.99	Not applicable
Binocular visual acuity (decimal)	0.96 ± 0.30	Not applicable
Reduced habitual acuity (<0.8)	OD: 21.4%; OS: 28.6%; OU: 21.4%	Not applicable
Refractive status (D)	OD: −3.25 to +4.00; OS: −3.00 to +2.50	Not applicable
Astigmatism (*n*)	OD: 5 players (−0.50 ± 0.17); OS: 5 players (−0.90 ± 0.91)	Not applicable
Stereopsis (Howard–Dolman, mm)	−4.37 ± 16.32	−39 to 46

Ranges are reported only for variables with clinically meaningful minimum–maximum values. For aggregated, categorical, or summary measures, ranges are not applicable.

**Table 2:** Within-subject comparisons of reaction-time variables (*n* = 28).

Comparison	First (Mean ± SD)	Second (Mean ± SD)	Test	Statistic	*p*-Value	Effect Size
E00 vs. E01	1.10 ± 0.52	1.28 ± 0.48	Wilcoxon	W = 50.5	0.130	r = 0.30
S00 vs. S01	0.41 ± 0.04	0.47 ± 0.07	Paired *t*-test	t(27) = −2.95	0.009	d = −0.69
E00 vs. E10	1.10 ± 0.52	0.92 ± 0.26	Paired *t*-test	t(27) = 1.14	0.270	d = 0.31
E00 vs. E11	1.10 ± 0.52	1.31 ± 0.38	Paired *t*-test	t(27) = −1.23	0.237	d = −0.32
S00 vs. S20	0.41 ± 0.04	0.55 ± 0.20	Wilcoxon	W = 23.0	0.011	r = 0.45
S00 vs. S21	0.41 ± 0.04	0.89 ± 0.43	Wilcoxon	W = 1.0	<0.001	r = 0.86
E10 vs. E11	0.92 ± 0.26	1.31 ± 0.38	Paired *t*-test	t(27) = −4.50	<0.001	d = −0.85
S20 vs. S21	0.55 ± 0.20	0.89 ± 0.43	Wilcoxon	W = 1.0	<0.001	r = 0.86

Paired comparisons were performed using paired *t*-tests when normality assumptions were met (Shapiro–Wilk *p* > 0.05) and Wilcoxon signed-rank tests otherwise. Effect sizes are reported as Cohen’s d for parametric tests and r (Z/√n) for non-parametric tests.

## Data Availability

The data presented in this study are not publicly available due to privacy and ethical restrictions, as they involve sensitive data collected from adolescent participants. Data supporting the findings of this study are available from the corresponding author upon reasonable request and with appropriate ethical approval.

## Supplementary Materials

The following supporting information can be downloaded at: https://www.mdpi.com/article/10.3390/s26082555/s1. Supplementary File S1: Ethics Committee approval (CEIm Hospital Clínico San Carlos, Approval ID: 23/415-E); Supplementary File S2: Informed consent forms for parents/legal guardians and adolescent participants; Table S1: Functional classification of reaction-time task codes; Figure S1: On-court implementation of the integrated visual–motor training drills; Figure S2: VR-based vergence training setup; Figure S3: Individual pre–post changes in a representative elective reaction-time condition; Figure S4: Individual pre–post changes in left-eye accommodative facility; Figure S5: Individual pre–post changes in D17 visual field reaction time.

## Abbreviations

The following abbreviations are used in this manuscript:

AC/A	Accommodative Convergence/Accommodation ratio
ANOVA	Analysis of Variance
cpm	Cycles per minute (accommodative facility frequency)
D17, D65, D86, D154, D192	Peripheral-field positions evaluated by the visual-field software
Hz	Hertz (frequency of stroboscopic stimulation)
NPC	Near Point of Convergence
NPA	Near Point of Accommodation
PPD	Pupillary distance (inter-pupillary distance), if applicable
SD	Standard Deviation
SPSS	Statistical Package for the Social Sciences
UFOV	Useful Field of View
VR	Virtual Reality
