# Supplementary material for: Sensor-Based and VR-Assisted Visual Training Enhances Visuomotor Reaction Metrics in Youth Handball Players
Source: Sensors (Basel). 2026 Apr 21;26(8):2555. doi: 10.3390/s26082555 (PMC13120118; doi:10.3390/s26082555)
Supplement: Supplementary file 1 [file sensors-26-02555-s001.zip › sensors-4116937-Suplementary Material-for-XML/Supplementary File S1.pdf]

Dictamen Protocolo Favorable

C.I. 23/415-E

28 de junio de 2023

Dra. Mar García Arenillas
Presidenta del CEIm Hospital Clínico San Carlos

CERTIFICA

- Que el CEIm Hospital Clínico San Carlos en reunión de Comisión Permanente, acta 7.1/23, ha evaluado la respuesta a las aclaraciones solicitadas con anterioridad al estudio:

Título: ***"BIOBANDING, ESTUDIO DE LA CONDICIÓN FÍSICA, COMPOSICIÓN CORPORAL Y VISUAL EN NIÑOS Y ADOLESCENTES DEPORTISTAS"***

Investigador principal: Ricardo Bernárdez Vilaboa. Facultad de Óptica de la U.C.M.

Código Interno: **23/415-E**

**Tipo Documento**	**Versión**
Protocolo	Vers. 2.0 junio de 2023
Hoja Información de Paciente - MENORES	Vers. 2.0 junio de 2023
Hoja Información de Paciente - PADRES	Vers. 2.0 junio de 2023

- Que en este estudio:
  - Se cumplen los requisitos necesarios de idoneidad del protocolo en relación con los objetivos del estudio y están justificados los riesgos y molestias previsibles para el sujeto.
  - Es adecuado el procedimiento para obtener el consentimiento informado.
  - La capacidad del equipo investigador y los medios disponibles son adecuados para llevar a cabo el estudio.
  - El alcance de las compensaciones económicas previstas no interfiere con el respeto de los postulados éticos.
  - Se cumplen los preceptos éticos formulados en la Declaración de Helsinki de la Asociación Médica mundial sobre principios éticos para las investigaciones médicas en seres humanos y en sus posteriores revisiones, así como aquellos exigidos por la normativa legal aplicable en función de las características del estudio.

- Que este Comité ha decidido emitir un **DICTAMEN FAVORABLE**.

- Que en dicha reunión se cumplieron los requisitos establecidos en la legislación vigente – Real Decreto 1090/2015 – para que la decisión del citado CEIm sea válida.

- Que el CEIm Hospital Clínico San Carlos tanto en su composición como en sus procedimientos, cumple con las normas de BPC (CPMP/ICH/135/95) y con la legislación vigente que regula su funcionamiento, y que la composición del CEIm Hospital Clínico San Carlos es la indicada a continuación, teniendo en cuenta que en el caso de que algún miembro participe en el proyecto o declare algún conflicto de interés no habrá participado en la evaluación ni en el dictamen de la solicitud de autorización del proyecto.

Presidenta	**Dra. M. García Arenillas**	Esp. Farmacología Clínica
Vicepresidente	**Dr. A. Marcos Dolado**	Esp. Neurología
Secretaria Técnica	**Dra. L. Cabrera García**	Esp. Farmacología Clínica
Vocales	**Dr. M. Carnero Alcazar**	Esp. Cirugía Cardiovascular
	**Dr. J.A. García Sáenz**	Esp. Oncología Médica
	**Dr. F.J. Martín Sánchez**	Esp. Urgencias
	**Dr. A.M. Molino González**	Esp. Medicina Interna
	**Dª. M.L. Pastor Alfonso**	Otras No Sanitarias
	**Dª. M. Peláez Agudo**	Atención Primaria
	**Dª. T. Peña Rollán**	Ldo. Derecho
	**Dª. M. Sáenz de Tejada López**	Farmacia
	**Dª. I. Serrano García**	Otras No Sanitarias (Exp. Estadística)
	**D. S. Varga Vázquez**	Enfermería
	**Dr. C. Verdejo Bravo**	Esp. Geriatría

Para que conste donde proceda, y a petición del promotor/investigador.

Lo que firmo en Madrid, a 28 de junio de 2023

GARCIA ARENILLAS MARIA DEL MAR - 05250249Q

Firmado digitalmente por GARCIA ARENILLAS MARIA DEL MAR - 05250249Q
Fecha: 2023.06.28 13:11:49 +02'00'

Fdo.: Dra. Mar García Arenillas
Presidenta del CEIm Hospital Clínico San Carlos
