# Supplementary material for: Sensor-Based and VR-Assisted Visual Training Enhances Visuomotor Reaction Metrics in Youth Handball Players
Source: Sensors (Basel). 2026 Apr 21;26(8):2555. doi: 10.3390/s26082555 (PMC13120118; doi:10.3390/s26082555)
Supplement: Supplementary file 1 [file sensors-26-02555-s001.zip › sensors-4116937-Suplementary Material-for-XML/Supplementary File S2.pdf]

Hoja de información al paciente

TÍTULO DEL ESTUDIO	
DISPOSITIVOS DE REALIDAD AUMENTADA UTILIZADOS EN PATOLOGÍAS OCULARES Y DE LA VÍA VISUAL. (DRAUPOVIVI)	
**CÓDIGO DEL ESTUDIO**	Código Interno: 23/614-E
**INVESTIGADOR PRINCIPAL**	**RICARDO BERNÁRDEZ VILABOA**
**CENTRO**	**Facultad de Óptica y Optometría. UCM**

Nos dirigimos a usted para informarle sobre un estudio de investigación en el que se le invita a participar. Nuestra intención es que usted reciba la información correcta y suficiente para que pueda decidir si acepta o no participar en este estudio. Para ello lea esta hoja informativa con atención y nosotros le aclararemos las dudas que le puedan surgir. Además, puede consultar con las personas que considere oportuno.

Participación voluntaria

Vamos a comprobar su estado visual. *Debe saber que su participación en este estudio es voluntaria y que puede decidir NO participar. Si decide participar, puede cambiar su decisión y retirar el consentimiento en cualquier momento, sin que por ello se altere la relación con su investigador ni se produzca perjuicio alguno en su atención sanitaria.*

Objetivo del estudio

-Analizar la Función Visual mediante dispositivos de realidad aumentada (gafas electrónicas para percibir la imagen en un visor de dimensiones muy pequeñas) utilizados en patologías oculares y de la vía visual.

Objetivos secundarios

-Caracterizar las pruebas visuales en una población con patologías oculares y de la vía visual.

-Parametrizar una ayuda de RA para hacer la Neurorrehabilitación Visual (que consiste en ejercicios para estimular áreas del cerebro poco utilizadas por varias pérdidas visuales a través de los ojos) en personas con patologías oculares y de la vía visual.

-Comparación de la Función Visual antes y después de la Neurorrehabilitación Visual en personas con patología ocular y de la vía visual.

Descripción del estudio

Vamos a buscar un mínimo de 100 participantes en patologías oculares y de la vía visual a evaluar y entrenar. No se van a utilizar fármacos y las medidas son no invasivas. Se realizan medidas visuales (Agudeza visual, refracción ocular, biomicroscopía, motilidad ocular, medida del contraste, medida del color, medida de la estereopsis, retinografía, aberrometría, topografía

corneal, habilidades visuales, pruebas específicas con Retiplus y campo visual fundamentalmente) Se realizará en una primera visita y posteriormente se le invitará para participar en un entrenamiento para la mejora de la percepción siguiendo una secuencia de 5 sesiones de una hora.

Riesgos y molestias derivados de su participación en el estudio

El único inconveniente del estudio consiste en la espera antes de la medida. Esto supone una espera máxima de hasta 30 minutos si se un investigador atiende a 2 pacientes por tramo horario en la jornada.

Posibles beneficios

La revisión visual completa y las variables que indiquen pérdida de rendimiento servirán al paciente con patologías oculares y de la vía visual para conocer sus problemas visuales y solventarlos de inmediato con gafas o lentes de contacto o ayudas de baja visión, incluidas las electrónicas, cuando sea necesario pero también con un protocolo individualizado de entrenamiento visual específico con gafas electrónicas. Es posible no obtener ningún beneficio directo para los participantes.

Confidencialidad

"De acuerdo con el Reglamento general de protección de datos (Reglamento EU 2016/679), además de los derechos de acceso, rectificación, oposición y cancelación de datos (Ley orgánica 15/1999 de protección de datos), también tiene derecho a limitar el tratamiento de datos y solicitar una copia o que se trasladen a un tercero (portabilidad) los datos que usted ha facilitado para el estudio. Para ejercitar sus derechos, diríjase al investigador principal del estudio. Así mismo tiene derecho a dirigirse a la Agencia de Protección de Datos si no quedara satisfecho/a.

Otra información relevante

Debe saber que puede ser excluido del estudio si el investigador del estudio lo considera oportuno, ya sea por motivos de seguridad, por cualquier acontecimiento adverso que se produzca por la medicación que esté tomando o porque consideren que no está cumpliendo con los procedimientos establecidos. En cualquiera de los casos, usted recibirá una explicación adecuada del motivo que ha ocasionado su retirada del estudio. Al firmar la hoja de consentimiento adjunta, se compromete a cumplir con los procedimientos del estudio que se le han expuesto.

Contacto en caso de dudas

Si durante su participación tiene alguna duda o necesita obtener más información, póngase en contacto con Ricardo Bernárdez Vilaboa, colaborador, profesor titular de escuela universitaria, Facultad de Óptica y Optometría, comunicación por teléfono 91 394 68 51.

Hoja de Consentimiento de Participante/CONSENTIMIENTO INFORMADO

Título del estudio	DISPOSITIVOS DE REALIDAD AUMENTADA UTILIZADOS EN PATOLOGÍAS OCULARES Y DE LA VÍA VISUAL. (DRAUPOVIVI)
**Código de protocolo**	*, fecha*

Yo, <<*nombre y apellidos del participante*>>

- ☐ He leído la hoja de información que se me ha entregado sobre el estudio.
- ☐ He podido hacer preguntas sobre el estudio.
- ☐ He recibido suficiente información sobre el estudio.
- ☐ He hablado con Ricardo Bernárdez Vilaboa
- ☐ Comprendo que mi participación es voluntaria.
- ☐ Comprendo que puedo retirarme del estudio:
  - Cuando quiera.
  - Sin tener que dar explicaciones.
  - Sin que esto repercuta en mis cuidados médicos.

Recibiré una copia firmada y fechada de este documento de consentimiento informado Presto libremente mi conformidad para participar en el estudio.

Firma del participante

Fecha: ____/____/____
(Nombre, firma y fecha de puño y letra por el paciente)

Firma del investigador

Fecha: ____/____/____

Cuando se obtenga el CI en personas con capacidad modificada para dar su CI

Firma del representante legal, familiar o persona vinculada de hecho

Firma del investigador

Fecha: ____/____/____
(Nombre, firma y fecha de puño y letra por el paciente)

Fecha: ____/____/____

Hoja de Consentimiento de Participante/CONSENTIMIENTO INFORMADO

**Título del estudio**	**DISPOSITIVOS DE REALIDAD AUMENTADA UTILIZADOS EN PATOLOGÍAS OCULARES Y DE LA VÍA VISUAL. (DRAUPOVIVI)**
**Código de protocolo**	*fecha*

Yo, *<<nombre y apellidos del participante>>*

- ☐ He leído la hoja de información que se me ha entregado sobre el estudio.
- ☐ He podido hacer preguntas sobre el estudio.
- ☐ He recibido suficiente información sobre el estudio.
- ☐ He hablado con Ricardo Bernárdez Vilaboa.
- ☐ Comprendo que mi participación es voluntaria.
- ☐ Comprendo que puedo retirarme del estudio:
    - Cuando quiera.
    - Sin tener que dar explicaciones.
    - Sin que esto repercuta en mis cuidados médicos.

Recibiré una copia firmada y fechada de este documento de consentimiento informado Presto libremente mi conformidad para participar en el estudio.

Firma del participante

Firma del investigador

Fecha: ____/____/____
(Nombre, firma y fecha de puño y letra por el paciente)

Fecha: ____/____/____

Cuando se obtenga el CI en personas con capacidad modificada para dar su CI

Firma del representante legal, familiar o persona vinculada de hecho

Firma del investigador

Fecha: ____/____/____
(Nombre, firma y fecha de puño y letra por el paciente)

Fecha: ____/____/____
