# Supplementary material for: Sensor-Based and VR-Assisted Visual Training Enhances Visuomotor Reaction Metrics in Youth Handball Players
Source: Sensors (Basel). 2026 Apr 21;26(8):2555. doi: 10.3390/s26082555 (PMC13120118; doi:10.3390/s26082555)
Supplement: Supplementary file 1 [file sensors-26-02555-s001.zip › sensors-4116937-Suplementary Material-for-XML/Supplementary Material.pdf]

Sensor-Based and VR-Assisted Visual Training Enhances Visuomotor Reaction Metrics in Youth Handball Players

Ricardo Bernárdez-Vilaboa, Juan E. Cedrún-Sánchez, Silvia Burgos-Postigo, Rut González-Jiménez, Carla Otero-Currás and F. Javier Povedano-Montero*

Table S1. Functional classification of reaction-time task codes

Code Prefix	Task Type	Cognitive Demand	Stimulus–Response Mapping	Motor Component	Description within Protocol
**S** (e.g., S00, S01, S20, S21)	Simple Reaction	Low	Single predefined stimulus–response pairing	Manual or foot activation	Participant responds to a single, predetermined visual stimulus without discrimination requirement.
**E** (e.g., E00, E10, E11)	Elective (Choice) Reaction	Moderate–High	Multiple stimulus–response options requiring discrimination	Manual or foot activation with directional movement	Participant must discriminate stimulus characteristics (e.g., color) and select the correct response before activation.
Numeric Suffix (e.g., 00, 10, 20, 21)	Spatial / Motor Variation	Not applicable	Reflects configuration differences	Return vs cross-over patterns	Indicates predefined spatial arrangement or movement execution variation within the drill sequence.

Functional classification and interpretative framework of reaction-time task codes generated by the instrumented reaction-light system. The table summarizes the structured coding system used to label reaction-time tasks within the experimental protocol. Code prefixes (e.g., S, E) indicate task type and cognitive demand (simple vs. elective reaction), while numeric suffixes denote predefined spatial configurations and motor execution variations. These identifiers were automatically assigned by the system to ensure traceability and reproducibility of stimulus–response conditions across baseline and post-intervention assessments.

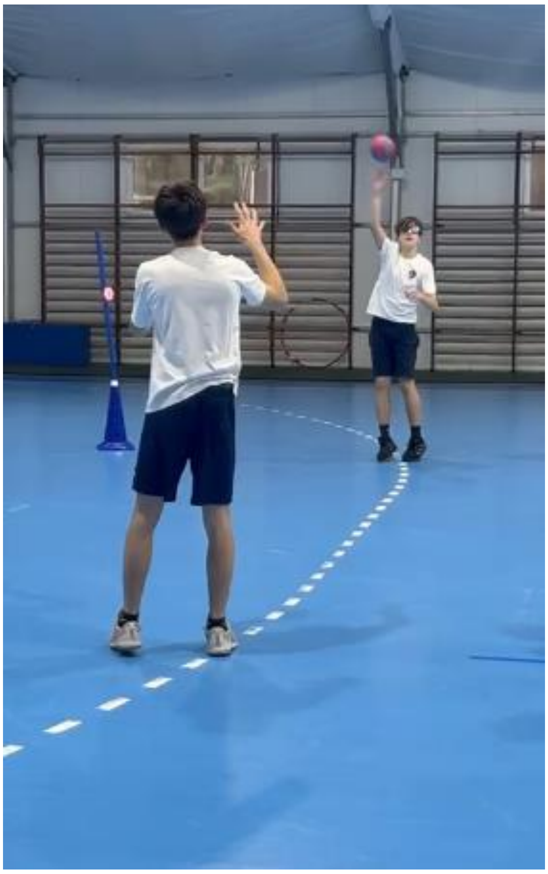

Figure S1. On-court implementation of the integrated visual–motor training drills. Example of the on-court implementation of the integrated visual–motor training drills during regular team practice. The image illustrates a representative reaction-light–based exercise performed in a real training environment, including rapid decision-making, passing, and motor execution. This figure highlights the ecological validity and practical integration of the protocol within routine handball training sessions.

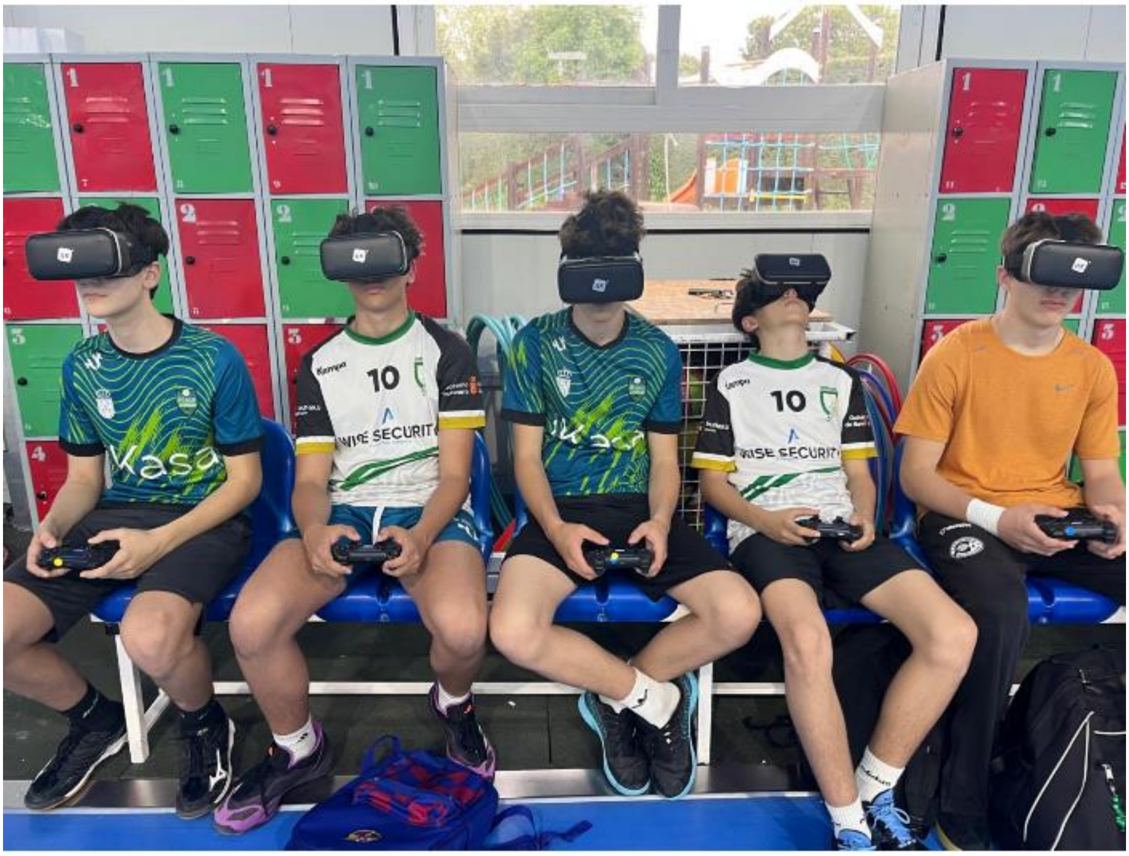

Figure S2. VR-based vergence training setup. Example of the VR-based vergence training component implemented during the integrated visual training program. Athletes performed binocular convergence and divergence tasks using a head-mounted display in a seated position under controlled conditions. Task difficulty was progressively adjusted across sessions by modifying disparity parameters to systematically stimulate vergence responses while maintaining stable head position and controlled viewing distance. This setup illustrates the practical implementation and ecological feasibility of VR-assisted visual training within the team training environment.

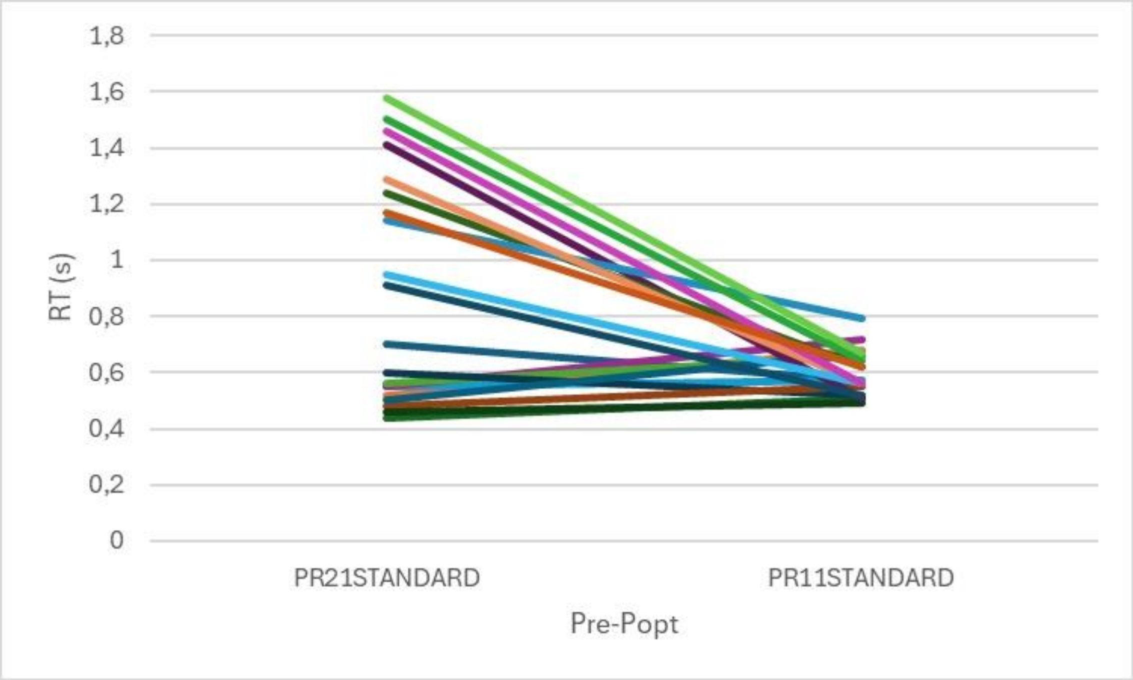

Figure S3. Individual pre–post changes in a representative elective reaction-time condition. Each line represents an individual participant, illustrating within-subject changes in reaction time (seconds) from pre-intervention (PR21STANDARD) to post-intervention (PR11STANDARD). This visualization highlights both the overall trend and inter-individual variability in response to the integrated visual–motor training program.

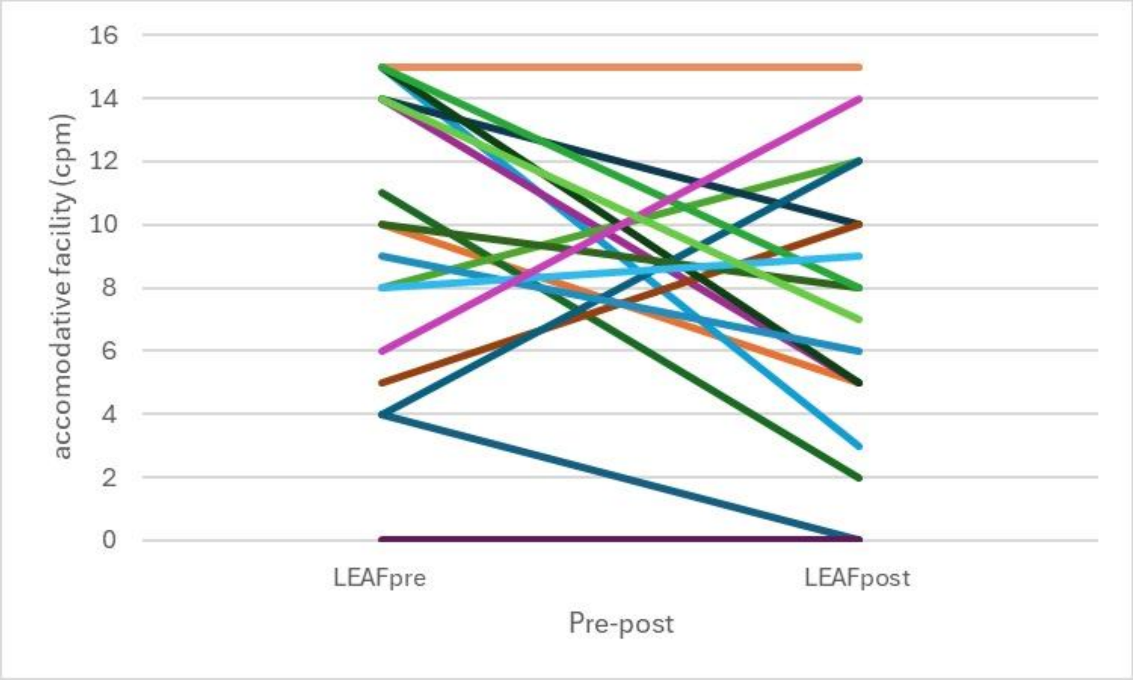

Figure S4. Individual pre–post changes in left-eye accommodative facility. Each line represents an individual participant, illustrating within-subject changes in accommodative facility (cycles per minute, cpm) from pre-intervention (LEAFpre) to

post-intervention (LEAFpost). This figure highlights inter-individual variability and the overall pattern of accommodative response following the integrated visual–motor training program.

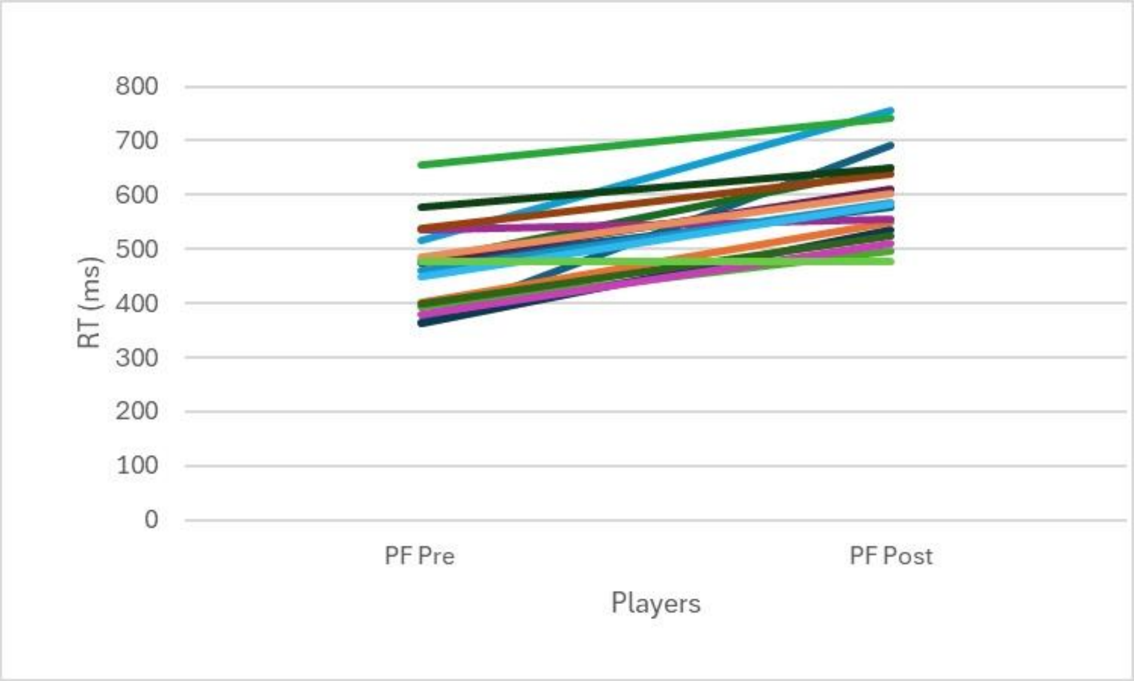

Figure S5. Individual pre–post changes in D17 visual field reaction time. Each line represents an individual participant, illustrating inter-individual variability and within-subject changes following the visual training program.
